# Every-Day Lessons from Every-Day Cases

**Published:** 1908-03

**Authors:** Charles A. L. Reed

**Affiliations:** Cincinnati, Ohio


### Every-Day Lessons From Every-Day Cases

By CHARLES A. L. REED, M. D., Cincinnati, Ohio

[*Lancet-Clinic*, November 16-30; December 14-28, 1907]

### No. 1

*"She thought it was her change of life."*

LET me present a familiar, an all too familiar, picture: a woman of forty-five or thereabouts enters the consultation-room. She is paler than a woman ought to be, her lips quiver a little, and a furtive glance of apprehension may be noticed in her eyes.

Her history is taken down—a healthy girl, a timely marriage, several children, natural deliveries, no illness of any sort—in short, a clean bill until about eight months ago. Then, instead of being unwell for a couple of days only, as had been her habit for the last year, she dribbled along for four days, then for a whole week.

Then she spoke about it to her neighbors. "It's only change of life; don't squander money a-goin' to the doctor; jes' take teas; I'll make some fer ye," was the reply from every wise old granny in the village.

One day, in spite of the teas, she had a hemorrhage and passed clots—a lot of them, and big ones, too. Then she did speak to the doctor; in fact, she sent for him.

"Oh, it's just the change of life; I'll fix it; here, take this," was his diagnosis, as he gave her, not an examination, but a wise look, a reassuring smile and some ergot.

Six weeks later she's no better; the ergot has ruined her digestion; she still bleeds, and she's getting weaker. Then she goes to the other doctor. He examines her—the very thing she wanted to have done from the start, only she was too timid to say so, and the first doctor failed in his duty by not suggesting it.

But now the dutiful doctor—the other one —examines her, looks stern and serious, and, possibly, there is a mingled expression of pity and hopelessness on his face. And it is there yet when, the very next day, he walks with his patient into the consultation-room of the surgeon. The patient herself is not only pale and tremulous, but she has an odor about her—a peculiar tell-tale odor.

The diagnosis is made then and there, but the history is taken and the examination is made with a feeling of solemnity. A look, a little nod of the head, and the surgeon has told the faithful attending physician, more eloquently than by words, that his diagnosis is confirmed.

It is cancer of the uterus! What is more, the cervix, soft and cheesy, with a crater in the center of it, occupies the whole vagina and bleeds when it is touched; the body of the uterus is immobile and hard masses are felt above the fornices. Then the finger is withdrawn, followed by a dark, ichorous, bloody, offensive discharge from the vagina. She's a doomed woman!

Who's responsible for having deprived her of the only chance she had, which, of course, was early in the disease? Every ignorant woman, who just knew that she knew all about it and that it "was change of life," and unhesitatingly assumed responsibility for the treatment of the awful condition, ought to be indicted for some sort of offense—manslaughter, or something as severely punishable at law.

And the first doctor, what of him? Is he not even more culpable? For he certainly ought to have known what his patient's story meant, and he ought to have acted on the knowledge. But this is a bootless inquiry. What is the lesson? Simply this: The "change of life"—the menopause—means, in health, only the decline and disappearance of the menstrual function—nothing more, nothing less. Any deviation from this rule, any hemorrhagic tendency, any capricious recurrence of the flow after it has once ceased, is an object of suspicion and calls for careful examination. In the majority of cases, possibly in nine out of ten, it means cancer. In the remaining cases it means a fibroid or a fungous endometritis or other neoplastic change. In all cases in which the condition is not obvious, microscopic investigation should be made. For in all these cases the time to act is at the earliest practicable moment.

The awful significance of the lesson I have been endeavoring to teach is being forced home on me almost daily, by case after case now on hand, parallel to the one I here cited, contrasted with many cases in which the diagnosis was made early, the operation was done promptly, and who are now well after periods varying from three years to eighteen years.

---

### No. 2

### *"Just Sour Stomach."*

"Why, Doctor, I thought it was just sour stomach," protested the fairly panic -stricken medical attendant when I arrived after a hurried motor ride of twenty and more miles. But let me tell the whole story as I got it.

Some eight or ten years ago a stalwart farmer of forty or thereabouts went to his physician. "Doctor, I've got an awful attack of sour stomach," he said. The diagnosis having been made for the doctor, the doctor evidently did not care to go beyond it.

Then, too, the probabilities were that it was correct. For there is more sour stomach in America than there are other functional disturbances of all the other organs of the human body combined. And generally it's the very simplest thing in the world to get rid of—at least for the time being. A purgative, a little bicarbonate of soda, with a restricted diet for a day or two, and the thing is done.

And so it seemed to be in the case of this good farmer. The treatment appeared to work, and the patient repeated it on his own responsibility from time to time—an example of the danger of telling your patients what you are giving them, however simple it may be.

But at more or less distant intervals an attack more severe than the preceding ones would send him back to the physician. At such times the sufferer would complain of a boring pain in the right upper quadrant, made worse by eating, and sometimes, an hour or two after meals, he would vomit his food, the emesis coming on suddenly and without premonitory nausea.

Then the doctor would look wise, give him some calomel tablets, and, by way of explanation, say: "Your secretions are perverted; here's something that will alter all that." Or on some other occasion the explanation would be modified: "You're bilious; I'll give you something to act on your liver," was the explanation. And then the patient got the same thing—calomel tablets—only the dose was a little larger, with possibly some alkali.

Several times his gums were tender, and once he had a free flow of saliva following this treatment. Of course, he knew nothing about the reaction of free hydrochloric acid in the gastric juice on the mild chloride of mercury. Nor did he know anything about the constitutional effects of the salt that was thus formed. So the doctor escaped without censure. And thus it went on for year after year, sometimes better, sometimes worse, until one time, after the leaves had fallen and the fodder was in the shock, the good patient, a little better than he had been for some time, went into the field with the boys to shuck some corn. He had been working for an hour or two, proud of his ability to make a full hand by the side of the husky youngsters, when he suddenly felt weak and paused. Then he went on again, shucked an ear or two, when he felt still weaker. Then came a sense of nausea, and he sat down by the side of the next shock.

A minute later his nausea increased and he vomited—yes, vomited, not only his partially digested breakfast, but great quantities of bright red, warm blood. He was too weak to call; for everything was first dim, then dark, and the sounds seemed far away. Then came oblivion.

"Do you know me, father?" were the first words that he heard. As he opened his eyes he saw his eldest son looking eagerly and tenderly into his face. "Where am I?" feebly enquired the stalwart man looking about him, alarmed to feel that he was bereft of his strength. "At home, dear," said the tender wife. "The boys brought you in. Now, now, you musn't talk. We've sent for the doctor; he'll be here soon!"

Then the poor man closed his eyes again; the pallor on his countenance increased; his lips seemed bluer than they were, and his hands were cold and clammy. But he was quiet—so quiet! "I'm sick," he suddenly cried, as he rallied his old-time strength and raised himself on his elbow. His eyes stared and he looked frightened. He emptied his stomach with a convulsive effort. A crimson stain was on the counterpane; the wall was spattered with blood, and so were the carpet and the chairs. The panic-stricken family, as they gathered about the great pool, were dumb with consternation.

The patient sank heavily on his blood-soaked pillow. He breathed quickly, then sighed—and did both while he slept.

The doctor arrived. "My goodness!" he exclaimed, "what's all this mean? The story was told as only such stories can be told at such times—told with great meanings crowded into little words and into looks that flashed their portent before even the little words could be spoken, told while the doctor searched for a pulse that he couldn't find. "We must have consultation; quick to the telephone!" commanded the doctor.

Then he went to work. The pillow was taken away; the foot of the bed was elevated; a messenger was despatched three miles for ice to put to the pit of the stomach; some adrenalin was permitted to trickle down the patient's throat and some was given hypodermatically; the emergency bag that the doctor always carried with him was brought out and a saline infusion was given within five minutes.

"Ah, he's coming!" exclaimed the doctor, as he again felt for the pulse and detected its flickering beats. Then for the next hour everything was done that the doctor could think of to coax the bleeding artery to stop its errant tide. The patient had rallied some and seemed hopefully restless. Then suddenly came another awful pallor, another awful gush, one that seemed to ensanguine everything and everybody. The pallor deepened into ashiness, the wild stare of the eyes became glassy under the drooping lids, the convulsive jaw relaxed, the last flicker of the pulse died away, and again the patient fell back, this time with a gurgle in his throat, to sleep beyond awakening.

Ten minutes later I arrived. When the story—the whole story—had been told to me by the distressed doctor I asked him: "How many attacks in all did he have during the years you had been treating him?" "Oh, probably fifteen or twenty bad ones; he was always grunting." "Did you at any time examine the stomach contents?" "No; I didn't think it was necessary: I thought he just had sour stomach."

"Did you ever wash out his stomach?"

"No; he seemed to get on pretty well on bicarbonate of soda."

"How extensive did you ever find the area of gastric resonance?"

"Why, I don't know's I ever looked for it?"

"Did you ever give him a rest-cure, with milk or cream diet?"

"No; I didn't think it was necessary; he always seemed able to get around; he always had a good appetite and liked 'most everything to eat."

"Then to-day is the first time this patient ever impressed you with the idea that he had an ulcer of the stomach?"

"Yes, I believe it is; once or twice I thought he might have it, but it all passed over."

As I was about to leave, the doctor led me to a more distant part of the dooryard, looked around, then stepped behind a cedar tree and looked again. Then he came a little closer, and speaking in a low, steady, earnest, almost pleading, voice, said:

"Doctor, I wish you'd go in and tell the family that I did everything that could be done."

---

### No. 3

*"Only the ignorant expect it;*
*Only fools wait for it."*

One winter's morning some twenty years ago I went at the usually early hour of seven o'clock to the late Mr. Lawson Tait's service at Spark Hill.

"What is the first case?" I asked while we were waiting for the anesthetist.

"You shall determine for yourself," was Mr. Tait's rather evasive reply.

Then, silent as the Sphinx, only with a smile on his well-chiselled face, he stood by while I made the examination.

The patient; a woman possibly thirty five, had a fluctuating, smoothly spherical mass, twice the size of a cocoanut, occupying and extending beyond the borders of the lower right quadrant of the abdomen; it retained its distinct contour when the patient was lying down; it could be moved up, down and sidewise, only its arc of mobility was limited; it could be felt through the fornix of the vagina and seemed to displace the uterus to the left; it was not painful on bimanual pressure; it had pushed the intestines upward and to the left. It had been about five months in course of development since it was first observed, then about the size of the patient's fist, but occupying its present locus; its further development had been without any marked or characteristic pain.

"It's a monocyst of the ovary," I exclaimed without a moment's hesitancy; nor was there any note of achievement in my voice, for, really, were not the conditions very obvious?

"It impresses me that way, too!" replied Mr. Tait, adding, somewhat meditatively, 'However, we shall see!"

A very few minutes later a few strokes of the knife had made an opening and two fingers were inserted into the peritoneal cavity.

"No, it is not ovarian," observed the operator *sotto voce.* Then he leaned a little over his patient and swept his fingers about while he closed his eyes in an instinctive effort to shut out all visual impressions and concentrate his attention on his tactile sense.

"The next probability," he continued to mutter, "is, of course, the gall-bladder!"

Another ten seconds of exploration and silence and he triumphantly exclaimed:

"Ah, I have it now; it's a large hydronephrosis." The rapidly-executed operation speedily confirmed the diagnosis. "Caused by the angulation of the ureter the result of displacement of the kidney" was the final explanation of the condition.

Then came the next case.

"Now," said Mr. Tait with a chuckle, "you were so successful in your diagnosis of the last case, suppose you try the next one."

I did. A slender young woman with a relaxed abdominal wall had a small tumor about the size of a mandarin orange. It was located just below the navel, and, possibly, a little to one side or the other of the median line, I have forgotten which. It was spherical, readily movable, and painless. It was not connected with the pelvic organs, and there was no functional disturbance to indicate that it was connected with any other organ.

I had, at that time, never either seen, read or heard of anything of the kind, so I frankly said:

"I'll give it up. What is it?"

"I'm sure I don't know any more about it than you do," replied the great surgeon, with equal frankness. "I know," he added, "that it's a growth, a morbid growth of some kind, one that ought not to be there. I have told the patient that if she will let me cut her open, look in and see what it is, I will take it out if I then think it ought to be taken out. So we shall shortly see." And we did.

In less than two minutes Mr. Tait had made an incision and was exploring with two fingers of his left hand. "A chylous cyst of the mesentery," was his simple announcement.

Then came silence, the silence of the Tait operating-room. The little cyst was brought up, emptied by a curved trocar, and dextrously enucleated. The duct was tied off close to the gut, a stitch was put into the peritoneal opening in the mesentery, and—but I didn't start out to tell about the operation at all.

My theme found the next instalment when, the operations for the morning over, I found myself by the side of Mr. Tait in his carriage on our way to the Priory. He had had a hard session in London the night before, and rather naturally pulled his tall hat down over his brows and almost disappeared in the ample recesses of his great fur collar. I thought he was trying to strike an average by stealing forty winks or so, when, suddenly, in half-muffled tones, he said: "Those were interesting cases—the first two."

"Interesting diagnostic studies," I assented, with rather a rueful recollection of my experience.

"Ah, that's just the point!" he exclaimed, as he suddenly emerged from the fur collar, restored his hat to the normal angle, sat upright, and with energy continued:

"It's wonderful how pretentious some people are about their diagnostic skill. You'd think they could look into and through one. It's almost comical to hear them guess and then watch their discomfiture when they open the case. One of your youthful Americans was over here not long since. He was one cock-sure sort of diagnostician. Out of four cases he missed it in three—yet he was cock-sure in all of them. They are cock-sure in London, too!"

There was a tone of irony in this remark, for Mr. Tait didn't like London, or, at least, many of the London surgeons.

"Now look at that kidney case!" he went on. "Every symptom was ovarian; not one pointed to the kidney. Then, too, the law of probability was a hundred to one on the side of the ovary. Then, too, it might have been a gall-bladder or a pelvic abscess—I've seen both in that location with an almost similarly confusing history. Yet, what did we find! And what would your fine cock-sure diagnostician have done with the other case? And suppose his diagnosis had been correctly made—correctly guessed—in both cases, what more indication for or against operation would he then have had than he had at first—what more would he have had before we made the incision?"

At this the vicissitudes of a London night seemed again to assert their consequences, and Mr. Tait again disappeared in his fur collar, from the depths of which, however, after a few minutes, he exclaimed: "I tell you, Reed, there is a lot of rubbish—yes, rubbish and worse—about much of our latter-day pretentiousness on diagnosis. Why, I see patients every day, certainly every week, who have been permitted to develop fatal complications while going from one wiseacre to another to get a hair-splitting diagnosis of conditions, the absolutely surgical character of which was obvious from the very start. Oh, it's tragic!"

"They are probably afraid of a diagnostic incision," I ventured.

"Afraid! Yes and no. 'Yes,' because they don't know, or at least don't realize, that right here in my hands, in the hands of any good surgeon—mind you, *good* surgeon—a mere exploratory incision is followed by no mortality whatever; 'no,' because the majority of them—the medical attendants—do not comprehend the proposition at all. They're still under the spell of the elders; they're still following the old diagnostic method. Think of the damage they are doing with their beatings and pokings and proddings and stabbings. Oh, it disgusts me!" At this Mr. Tait kicked the foot-warmer against the front of the carriage and swore because he hurt his toe.

"When you go back to America," he resumed, after he had regained the thread of his discourse, "preach it—yes, preach it and preach it fearlessly—that an exploratory incision is a legitimate diagnostic measure. Don't mince words. Say even more. Say that in certain cases the man who doesn't have recourse to it, but trifles his patient's chances away while waiting for 'rational' signs to develop, is himself irrational—yes, worse; he is culpable. I am preaching the exploratory incision, and practising it, too, as you saw this morning. I'm doing it right here in England, to the horror of the elders. And here, you know, it's a capital offense to perpetrate a new idea without the previous consent of the elders. But the dictum of the elders to the contrary notwithstanding, exploratory incision is a valuable principle—valuable in general surgery, invaluable in abdominal surgery. Many gastly blunders and no end of suits for malpractice will be saved by its general adoption in both fields. Absolute accuracy of diagnosis in the abdomen is very far from being possible. Only the ignorant expect it; only fools wait for it." Just then we arrived at the Priory.

---

**No. 4**

*"Wise in his own conceit."*

Over in the other part of town—the part where the habitants abided—lived the judge whom and whose family—a wife and two fine, manly boys—we had come to know quite well during our summer's sojourn. So, when the sun began to drop into the lake a degree or so farther south each night, when the tinge of frost was felt in the Northern air, and we were about ready to start home, we went to make a farewell call. It was just at nightfall, and the judge—an athletic, strong-voiced, masterful man, once center on his college team—met us at the door.

"Come right in," he said in his hearty way. "Excuse my good wife for a little while; she's up stairs nursing one of the boys. And the servants are up there with her," he added, as if to explain why he himself had answered the bell.

"Nothing serious, I hope?"

"Oh, no; nothing. You know how mothers are. If they had their way about it they'd make molly-coddles of all their boys."

This, addressed more to my good wife than to myself, was said in a spirit of intended raillery, but with a reserve note of earnestness that gave to his would-be humor the ring of the counterfeit.

"Why do you think so?" she discreetly replied.

"Well, take this instance: Here's Frank, a strapping boy of fifteen—shall we not sit here in the shadows? Yes, I like it better myself—well, as I was saying, he's a strapping fellow, never been ill a day in his life, positively not a single day. Last Tuesday he was out in the country, and, boy-like, gorged himself with a lot of green apples. That night he had what I fancy all of us have had at one time or another, and probably from the same cause—a case of old-fashioned cramps. His mother was at once alarmed, and if he hadn't been a cool-headed boy she would have alarmed him, too. Of course, she wished to send for a doctor right away. But I put my foot down on that proposition. You see, I don't believe in calling doctors for trivial ailments." This, with a chuckle of good humor that was hardly sufficient to conceal the wire edge of prejudice, was addressed to me.

"Yes," I admitted, "doctors are often cheated out of their rest by unnecessary calls."

"Just so! Besides, I know a thing or two myself about ills and remedies. Most ills are imaginary, and even when they are not imaginary, nature—nature, I say—is their best remedy. Then, too, spirit, pluck, has a lot to do with it!"

The judge's face was wreathed in a smile of self-satisfaction that dissipated the deepening gloom of evening as he mentally reviewed this learned disquisition and paused as if for applause. But before the hand-clapping could begin he continued: "So, when I found Frank inclined to loaf around I made him sit up, even against his mother's protest. That was three or four days ago. Of course, his mother protested. But yesterday I simply made him get up and go to the baseball game. You see, Frank is fond of baseball, and I knew if he got interested in the game he'd forget to grunt."

I thought I scented a family altercation back of this incident, but I simply asked: "How did it work?"

"Oh, why—pretty well. He complained some, lay down on the benches once, but I soon shamed him out of that. He was a little upset at his stomach on his way home, but after the roasting I had given him he never whimpered. Game, wasn't he?"

"He must have lots of grit," I assented, hoping that there might be an end of the narrative.

"His mother tried to make me believe that he had a chill after he got home, but it was kind o' cool last night, anyhow. Then she said he had fever. This morning I went in to see him before I went to court. His hands and feet were cool, not a bit feverish, and he said he guessed he'd soon be all right. His pain was all gone. But his mother says he's worse in spite of all this, and she's made such a fuss that I have sent for Doctor Hopestock. By the way, do you know him?"

"Yes, very well; a clever practitioner. You can take his advice implicitly," I said as we arose to go, intimating that we might be detaining him from duties upstairs.

"No, no; don't go! I'm not needed at all. Besides, the doctor will be here in a few minutes, and when he is through we can have a nice chat."

While we were gently protesting our host exclaimed: "Ah, there he comes now. Just wait a minute." With this he met the doctor at the door and escorted him upstairs. Five minutes later the judge came down. For the first time his air of self-satisfaction gave way to a look of perturbation. "The doctor wishes you to come up stairs at once, if you will be so kind," he said to me.

The doctor, a man of unusually excellent poise, seemed in a state of alarm as he met us at the head of the stairway. "This is awful!" he said, as he ushered me along.

And there lay Frank, as handsome and manly a looking fellow as I had ever seen. But his nostrils were pinched, his lips were more blue than red, and there was a pallor on his cheeks. His respiration was shallower and more humid than normal. There was a mawkish sweet odor to his breath. His hand, even his wrist, was cool and humid and his pulse beat 140 to the minute. Just then the doctor took his thermometer from under the patient's axilla and found that it registered 105°. The abdomen, bared, was found distended and tympanitic.

"Does it hurt there?" I asked as I gently tapped the lower right quadrant.

"No; that's where all the pain was, but it don't hurt a bit today," replied the lad with panting respiration.

"That's what I told you, Doctor," interrupted the father with some impatience. "He's been better all day! I don't see any sense in all this alarm."

The mother, sitting bowed by the side of her manly boy, exchanged no look with her husband. We—the doctor and myself—retired for the totally unnecessary consultation. Agreement was reached by a glance and a nod. The father was summoned.

"Well," demanded he with some impatience, "what is it?"

Appendicitis," answered Dr. Hopestock, modestly, almost with bated breath.

"Appendicitis? Nonsense! It's bellyache! You doctors have gone crazy about appendicitis!" roared the apparently irate parent.

"I beg your pardon," I interposed, "but may I correct Dr. Hopestock?"

"Certainly, Dr. Reed, certainly," the judge rather eagerly assented, with a glance of triumph toward his medical attendant.

"It 's death!"

"What! What do you say?"

"I say it is death—death due to appendicitis!"

"Do you mean to tell me that that boy is going to die?"

"Within the next twenty-four hours!"

The man, wise in his own conceit, still denied and defied. "I would not do my whole duty here," I said, "unless I told you the whole truth. Your son has appendicitis; he had it last Tuesday; he needed this good physician then; he has needed him every minute from that time until within the last few hours; since then it has been unnecessary. You have ignored the just claim of your wife and the ordinary human necessities of your son for relief. Your treatment of him has been not only cruel, but homicidal. Save for the fact that he might have died anyhow, his blood is on your hands." The steel entered.

The proud man trembled—trembled in silence. A rustling noise attracted my attention, and turning I saw the form of the wife and mother swiftly glide from the shadow of the room into the deeper darkness of the hallway. She had heard it all!

After a minute the judge broke the silence. Placing a hand on each of our shoulders, he implored us to do something, and his pleadings only doubled when we explained that an abscess had formed and burst into the peritoneal cavity; that pus had absorbed from it into the circulation; that the patient was already practically dying from blood poisoning; that the only thing that could be done would be to open the abdomen and wash out the pus, but that even that would avail only as if by a miracle.

"If there's a thousandth part of a chance, for God's sake give it to my boy!"

In very pity the plea was granted. Within but little more than an hour the abdomen had been opened; quantities of pus ran out; the appendix had rotted away; the cavity was flushed with normal salt solution; the patient was given a salt infusion into his veins; he was given nitroglycerine and strychnia and put to bed with heat about him. He rallied from the operation.

But his pulse kept on getting faster and his breathing shallower. At ten the next morning he died.

The father wept. The mother, dry-eyed, clasped her hands and gazed into vacancy.

"Frank! my Frank!" she gently murmured. The next year, when we went back, we learned that she had been taken to the asylum, where, walking the long corridors, she still gazed into vacancy and still gently murmured:

"Frank! my Frank!"

And Dr. Hopestock said in tones of discouragement: "I've had another case since then. I saw it early. An operation was clearly necessary and the conditions were all favorable. The family and patient had agreed to send for a surgeon. Just then our friend, the judge, appeared upon the scene. 'Don't have an operation?' he exclaimed. 'They operated on my boy and he died. I don't believe in operations.' They took his advice as against mine. Of course, the patient died. So I have lost both of my cases."

"Yes," said I, "and one man killed both of them—and that man was not you. They were killed by a man who was wise in his own conceit."

